# Recent Developments of Nanostructures for the Ocular Delivery of Natural Compounds

**DOI:** 10.3389/fchem.2022.850757

**Published:** 2022-04-13

**Authors:** Malihe Sadat Razavi, Pedram Ebrahimnejad, Yousef Fatahi, Antony D’Emanuele, Rassoul Dinarvand

**Affiliations:** ^1^ Department of Pharmaceutics, Faculty of Pharmacy, Mazandaran University of Medical Sciences, Sari, Iran; ^2^ Nanotechnology Research Centre, Faculty of Pharmacy, Tehran University of Medical Sciences, Tehran, Iran; ^3^ Pharmaceutical Science Research Center, Hemoglobinopathy Institute, Mazandaran University of Medical Sciences, Sari, Iran; ^4^ Leicester School of Pharmacy, De Montfort University, Leicester, United Kingdom

**Keywords:** nanotechnology, nanoparticles, natural products, ocular drug delivery, eye

## Abstract

Ocular disorders comprising various diseases of the anterior and posterior segments are considered as the main reasons for blindness. Natural products have been identified as potential treatments for ocular diseases due to their anti-oxidative, antiangiogenic, and anti-inflammatory effects. Unfortunately, most of these beneficial compounds are characterised by low solubility which results in low bioavailability and rapid systemic clearance thus requiring frequent administration or requiring high doses, which hinders their therapeutic applications. Additionally, the therapeutic efficiency of ocular drug delivery as a popular route of drug administration for the treatment of ocular diseases is restricted by various anatomical and physiological barriers. Recently, nanotechnology-based strategies including polymeric nanoparticles, micelles, nanofibers, dendrimers, lipid nanoparticles, liposomes, and niosomes have emerged as promising approaches to overcome limitations and enhance ocular drug bioavailability by effective delivery to the target sites. This review provides an overview of nano-drug delivery systems of natural compounds such as thymoquinone, catechin, epigallocatechin gallate, curcumin, berberine, pilocarpine, genistein, resveratrol, quercetin, naringenin, lutein, kaempferol, baicalin, and tetrandrine for ocular applications. This approach involves increasing drug concentration in the carriers to enhance drug movement into and through the ocular barriers.

## Graphical Abstract

Application of nanostructures in ocular delivery of natural products.
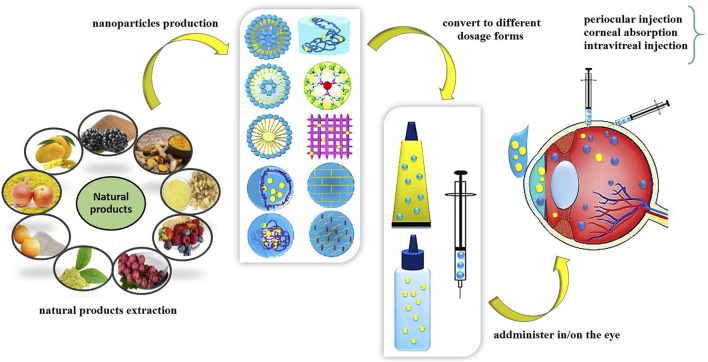

## 1 Introduction

**FIGURE 1:**
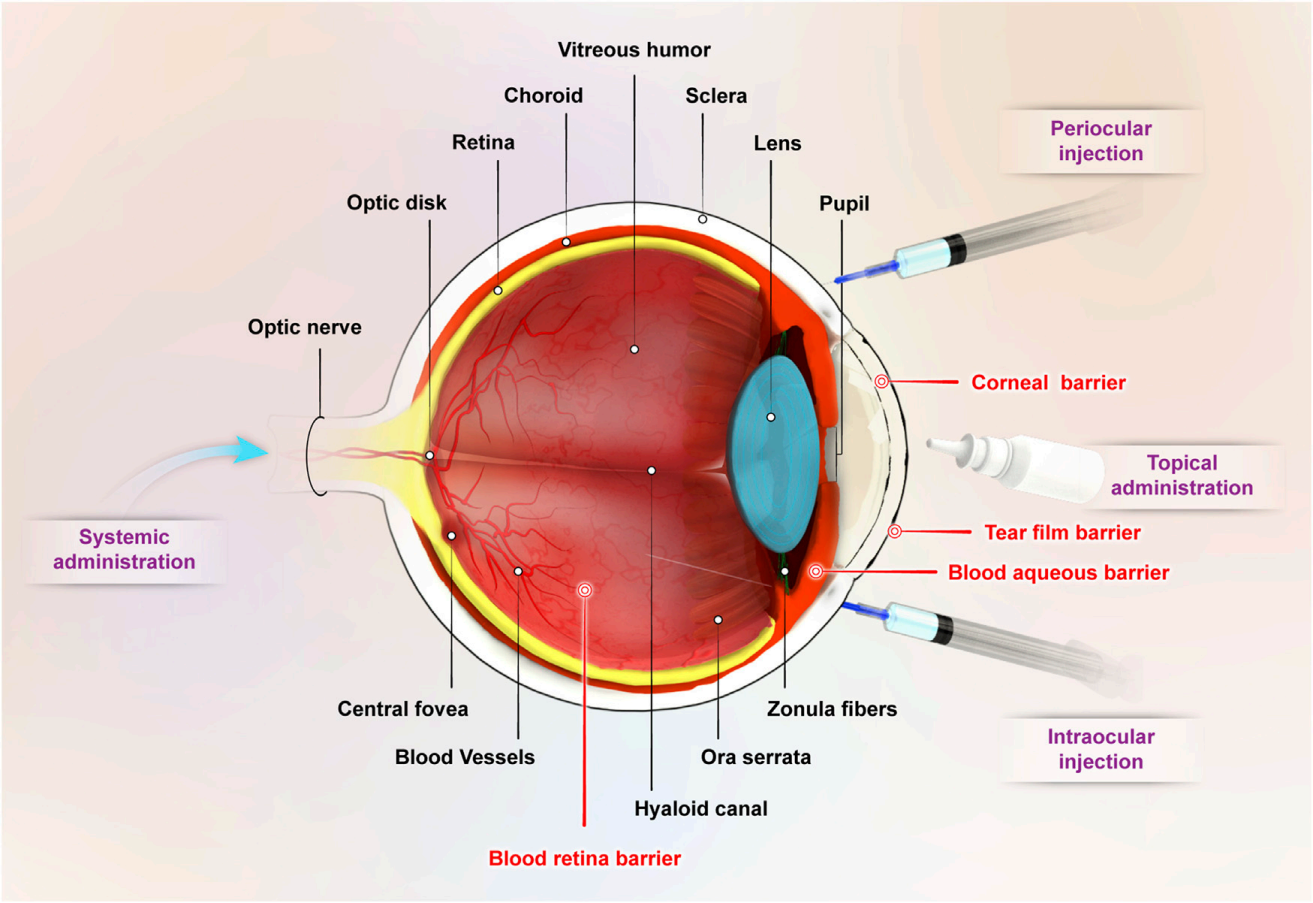
Ocular structure and main routes of ocular drug administration.

The anatomy and physiological characteristics of the eye makes it a unique sensory organ. The eye can commonly be categorized into two main compartments: the front one-third of the eye between the cornea and the lens which is called the anterior segment and the back two-thirds of the eye from the lens to the optic nerve, including the vitreous humor which is called the posterior segment (Figure 1). There are various chronic and acute diseases that can affect the anterior and posterior segments of the eye. The most common chronic posterior segment diseases such as age-related macular degeneration (AMD), diabetic macular edema (DME), and diabetic retinopathy (DR) and some of the chronic anterior segment diseases such as glaucoma, uveitis, cataract, dry eye syndrome (DES) are the leading causes of vision loss (Joseph and Venkatraman, 2017). Most eye diseases are associated with aging, oxidative stress mechanism, and inflammatory responses. While natural products with the ability to scavenger reactive oxygen species (ROS) and suppress inflammatory mediators can be considered as a promising remedy for the prevention and treatment of ocular diseases.

**FIGURE 2:**
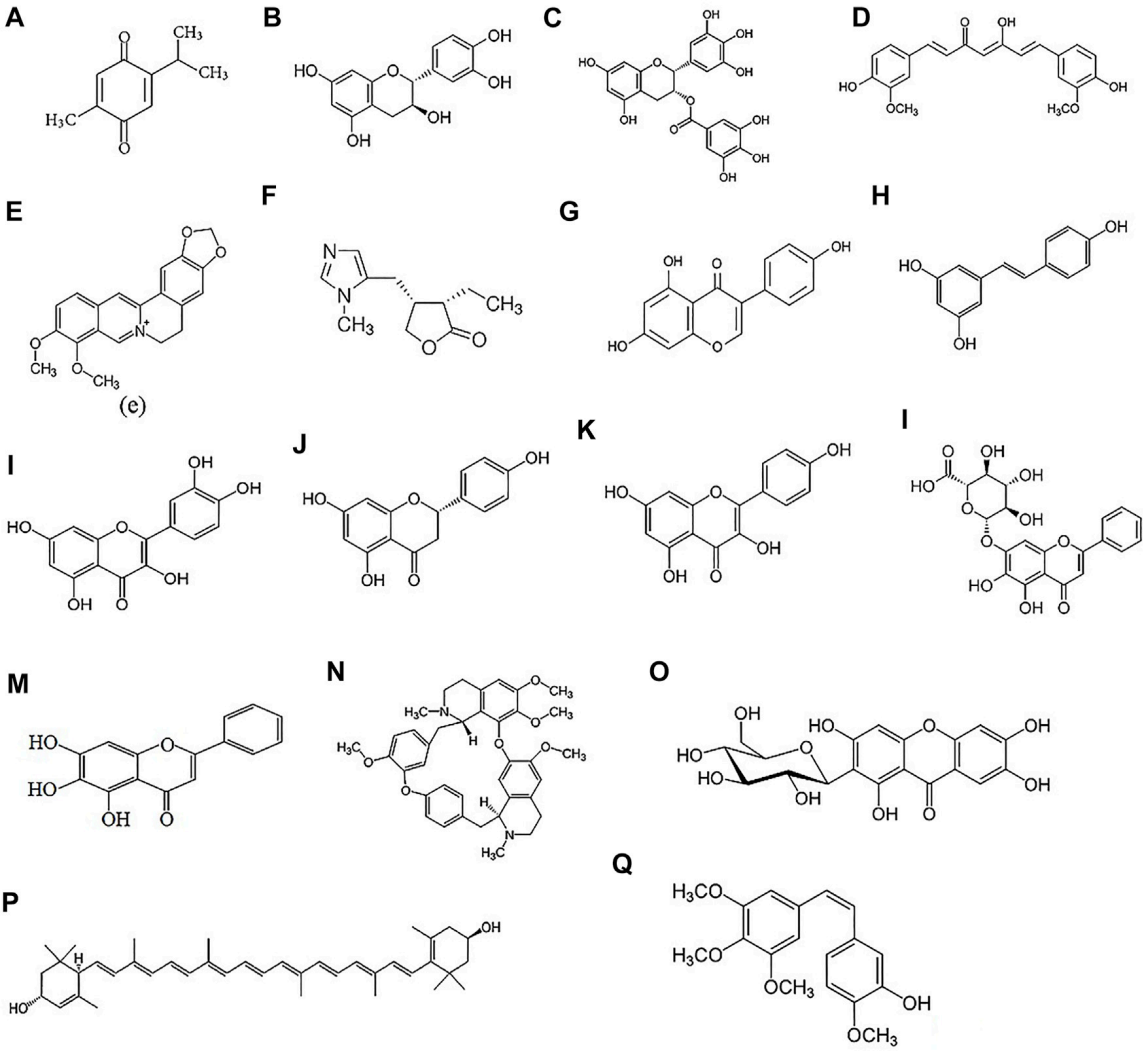
Chemical structure of natural products: Thymoquinone **(A)**, Catechin **(B)**, Epigalloctatin gallate **(C)**, Curcumin **(D)**, Berberine **(E)**, Pilocarpine **(F)**, Genistein **(G)**, Resveratrol **(H)**, Quercetin **(I)**, Naringenin **(J)**, Kaempferol **(K)**, Baicalin **(L)**, Baicalein **(M)**, Tetrandrine **(N)**, Mangiferin **(O)**, Lutein **(P)**, Combretastatin A4 **(Q)**.

Since early times, natural products containing active pharmacological ingredients with various molecular structures have been used for the treatment of numerous diseases and disorders. Many of these compounds possess strong anti-oxidative, anti-inflammatory, and anti-apoptotic effects. Natural products have a particular chemical and structural diversity (Figure 2), with less toxicity and currently, many of the modern drugs in use have their origin in natural compounds and their derivatives (Mathur and Hoskins, 2017). The alkaloids, flavonoids, and phenolic compounds are among the bioactive ingredients that exist in natural products. The chemical structures of natural compounds play a crucial role in their therapeutic effects and their biological properties. For example, lutein with two hydroxyl groups can effectively scavenge the free radicals and prevent the oxidation process, so it can be considered as a potential drug for the prevention and treatment of a posterior segment of the eye such as AMD (Koushan et al., 2013) or polyphenolic structure of curcumin makes it a promising candidate for the treatment of bacterial infection and inflammation (Gupta et al., 2012). Other studies also revealed that the replacement of the methoxy groups of curcumin with other groups change its anti-inflammatory effect and reduce this effect. These findings demonstrated the role of the aryl group of curcumin in its anti-inflammatory effect (Noureddin et al., 2019). Several studies have been performed to find herbal active ingredients such as curcumin, catechin, lutein, ginseng, resveratrol, quercetin, and many more to prevent or ameliorate sight-threatening eye diseases (Fathi et al., 2017; Kim et al., 2020). However, these compounds show low absorption ability, bioavailability, and efficiency due to their high molecular weight to pass through lipid membranes. However, the mechanisms of action of these ingredients are not fully investigated and few literature studies exist on the efficiency of natural compounds on human eye diseases, but there are many reasons to consider natural products that can work synergistically to enhance the activity of other drugs (Sulaiman et al., 2014).

The eye is a well-protected organ in the body and has several protective barrier layers and complex structures with various defense mechanisms that defend it against harmful substances, microorganisms, and toxins. These barriers that are essential in protecting and preserving vision also restrain the entry and penetration of drug molecules to the inner ocular tissues (Joseph and Venkatraman, 2017). Thus ocular drug delivery remains a great challenge to researchers and ophthalmologists due to the presence of these complex barriers. There are various pathways for drug delivery to the anterior and posterior segments of the eye including topical, periocular, systemic, and intravitreal routes (Figure 1) (Varela-Fernández et al., 2020). The advantages and disadvantages of these routes are summarized in Table 1. Conventional delivery systems such as eye drops, injections, and implants have been the most extensively utilized, but they have some disadvantages including challenges to traverse the physiological barriers, enzymatic drug degradation, protein binding, poor targeting efficiency, low penetration and retention time, side effects and low bioavailability (Ingle et al., 2017; Sánchez-López et al., 2017). Numerous researchers have attempted to develop non-invasive, cost-effective, sustained release approaches with enhanced therapeutic efficacy over conventional systems. Due to the complex structure and ocular barriers, there is a need for the rational design of drug delivery carriers to provide effective treatment. Nanocarrier-based drug delivery systems are designed to deliver the drug to the target site by delivering small molecules either by improving their permeation or by extending residence time, prolonging the drug release profile, and reducing the injection frequency (Shen et al., 2015). Several nanomaterials have been explored to overcome ocular barriers and control the release of drugs (Ingle et al., 2017; Lakhani et al., 2018a). Even with the few available reports about the delivery of natural products, nanoparticles as promising carriers could entrap these natural products to make a safe and more promising alternative for the remedy of ocular diseases through the ocular routes. This review highlights the various challenges associated with drug delivery to the anterior and posterior segments of the eye, and provide an overview of novel nanomaterials with the potential for ocular delivery of natural products, and treatment of ocular diseases.

**TABLE 1:** Routes of administration and their benefits and profits.

Routes of Administration	Advantages	Disadvantages
Topical	Easy formulation	Frequent administration
	Patient-friendly (easy to apply)	Rapid wash out
	Efficient for anterior segment disorders	Difficulties to reach the posterior site
	Safe to use	Poor bioavailability
		Low retention time on the eye surface
		Limited volume of dosage form
		Restricted by corneal barrier and tear film barrier
		Blurred vision (ointment form)
Pre-ocular	Less-invasive (in comparison with intravitreal injection)	Low bioavailability
	Minor side effects	Restricted by ocular barriers
	Efficient for posterior segment drug delivery	Rapid wash out
	Deliver high amount of drugs to the target site	Risk of drug degradation
	Long duration of action	
Intravitreal	Directly deliver the drugs to the posterior segment	Repeated injections
	Most efficient treatment for posterior segment	Invasive
		Numerous side effects
Systemic	Effective for the treatment of both anterior and posterior segments	Systemic side effects
		Restricted by blood ocular barriers (BRB and BAB)

## 2 Ocular Diffusional Pathways for Ocular Drug Delivery

**FIGURE 3:**
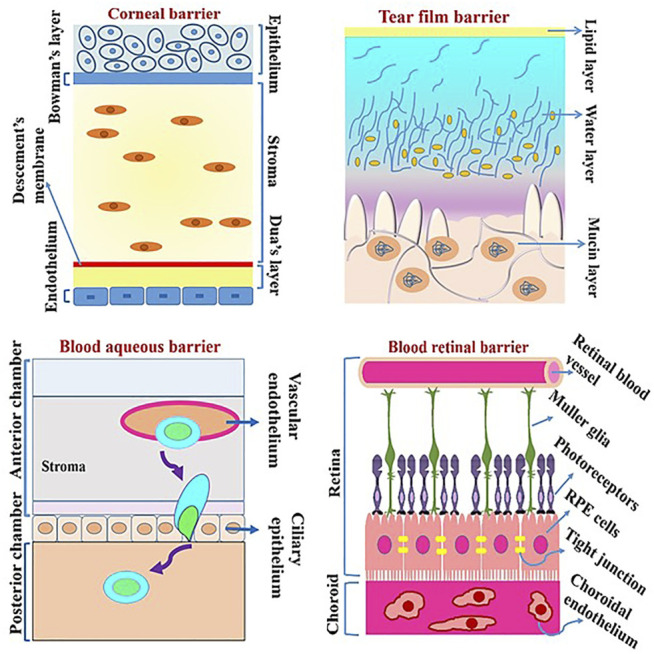
Various barriers in the eye which drugs must overcome to reach the target sites.

The anatomy and structure of eye are complex with two principle pathways for drugs to pass into ocular tissues and reach their target, namely the corneal and the conjunctiva-scleral (non-corneal) pathways. Drugs traversing each pathway encounter barriers such as pre-corneal (tear film), the cornea, the blood aqueous barrier (BAB), and the blood retinal barrier (BRB) (Figure 3) (Loftsson and Stefánsson, 2017). For easy reading abbreviations are listed in Table 2.

**TABLE 2:** List of abbreviations. For easy reading abbreviations are listed in TABLE 2.

Meanings	Abbreviations	Meanings	Abbreviations
cyclodextrins	CD	tetrahydrocurcumin	THC
thymoquinone	TQ	dry eye disease	DED
Intraocular pressure	IOP	latanoprost	LAT
poly (ethylene glycol) (PEG	PEG	blood-retinal barrier	BRB
dry eye syndrome (DES	DES	blood aqueous barrier (BAB	BAB
age-related macular degeneration	AMD	diabetic macular edema (DME	DME
vascular endothelial growth factor	VEGF	poly-ε-caprolactone	PCL
streptozotocin	STZ	posterior capsular opacification	PCO
Naringenin	NG	Genistein	GEN
retinal pigment epithelium degeneration	RPE	chrysophanol	CHR
choroidal neovascularization	CNV	curcumin	CUR
quercetin	QUR	Berberine	BBR
baicalin	BN	renal pigment epithelium-derived factor	PEDF
polyamidoamines	PAMAM	Kaempferol	KA
human umbilical vein endothelial cells	HUVEC	macular degeneration	MD
Soluplus micelle of resveratrol	SOL-RES	tetrandrine	TET
nanostructured lipid carriers	NLCs	gelatin nanoparticles	GNP
Lipid nanoparticles (LNPs)	LNPs	poly (epsilon-caprolactone)	PCL
Carboxymethyl chitosan	CMC	polylactic acid	PLA
genipin	GN	rhodamine 6G	Rh6G
confocal laser scanning microscopy	CLSM	nanocapsules	NCs
solid lipid nanoparticles	SLNs	nanospheres	NSs
electroretinogram	ERG	poly (alkyl cyanoacrylate)	PACA
blood retina barrier	BBB	poly (lactic-co-glycolic acid)	PLGA
encapsulation efficiency	EE	Resveratrol	RES
acrylic acid	AA	dimethyl dioctadecyl ammonium bromide	DDAB
riboflavin	RB	cetyltrimethylammonium bromide	CTAB
sodium alginate	SA	reactive oxygen species	ROS
rhodamine B	RhB	polymeric nanoparticles	PNPs
hyaluronic acid	HA	tripolyphosphate	TPP
oil/water	o/w	silk fibroin nanofibers	SFNF
polypropylimines	PPI	arginine–glycine–aspartic acid	RGD

### 2.1 The Corneal Pathway

#### 2.1.1 The Pre-corneal Barrier (Tear Film Barrier)

The main component of the pre-corneal barrier is through tear drainage (Ghate and Edelhauser, 2006; Gaudana et al., 2010). The conjunctival *cul-de-sac* can accommodate the low amount of topically administered eye drops (Lakhani et al., 2018a). Pre-corneal drainage causes the removal of the applied formulation and decreases the corneal residence time of the formulation (Nejima et al., 2005). In addition, tear fluid proteins can bind to the drug and lead to a reduced concentration of free drug in the tear fluid (Svitova and Lin, 2010).

#### 2.1.2 The Corneal Barrier

The cornea is a transparent multilayered barrier limiting drug penetration into the aqueous humor through the corneal pathway. It is comprised of five-layers with alternating lipophilic and hydrophilic characteristics. The lipophilic epithelium permits the diffusion of particles with dimensions up to approximately 20 nm (Yellepeddi and Palakurthi, 2016; Bisht et al., 2018). Stroma is hydrophilic in nature and only hydrophilic molecules up to 500 kDa of size are amenable to diffusion, while the entry of most hydrophobic drugs is restricted (Yellepeddi and Palakurthi, 2016). Thus, for efficient permeation across the cornea both molecular weight and logP should be optimized. The leaky corneal endothelium provides minimal resistance to the movement of macromolecules between the stroma and aqueous humor (Cheruvu et al., 2008; Yellepeddi and Palakurthi, 2016). The two principal routes for drugs to cross the epithelium are transcellular and paracellular pathways. Generally, lipophilic drug molecules permeate *via* the transcellular route whereas hydrophilic molecules and small ions pass through the paracellular route (Raghava et al., 2004).

### 2.2 The Conjunctival–Scleral (Non-corneal) Pathway

#### 2.2.1 Blood Aqueous Barrier

Topically administrated drugs may also pass through the conjunctiva and the sclera water channels/pores (ranging between 30 and 300 nm in size) facilitated by passive diffusion to get into the vitreous humor (Kompella et al., 2010). The conjunctiva is moist and highly vascularised epithelial tissue, so a significant amount of the drug molecules permeating through the conjunctival epithelium is eliminated *via* systemic absorption resulting in low drug bioavailability (Prausnitz and Noonan, 1998; Pearce et al., 2015; Battaglia et al., 2016).

#### 2.2.2 Blood Retinal Barrier

The choroid and especially Bruch’s membrane are considered as important barriers for the penetration of drugs. The blood retinal barrier (BRB) is a significant barrier composed of tight junctions between the retinal endothelial blood vessel and retinal pigment epithelium (RPE) cells (Jo et al., 2019). The RPE acts as a rate-limiting permeation barrier particularly to hydrophilic molecules where the time of permeation increases with increasing the molecular weight (Mains and Wilson, 2013).

## 3 The Importance of Natural Products in Ocular Diseases

The beneficial therapeutic effects of natural products has attracted a significant attention in the treatment and prevention of diseases. They are considered effective agents for the treatment and prevention of various ocular disorders such as glaucoma, cataract, corneal and choroidal neovascularization, AMD, DR, DES (Radomska-Leśniewska et al., 2019). Besides their beneficial effects, most of these natural products suffer from low water solubility and low bioavailability due to rapid enzymatic degradation that hinder their medical applications (Chebil et al., 2007; Khan et al., 2015; Shim et al., 2019). Different techniques have been applied to overcome these limitations such as encapsulating flavonoids into polymeric carriers, covalent conjugation of flavonoids to hydrophilic polymers, such as dextran (Yee et al., 2017), poly (ethylene glycol) (PEG) (Liang et al., 2018), poly (allylamine), and gelatin (Spizzirri et al., 2009). The focus in this section will be on natural products that have been delivered using nanotechnology systems in the area of ophthalmology. The chemical structures of these compounds is depicted in Figure 2. The characteristics, properties and applications of various natural products used in ocular drug delivery are summarised in Table 3.

**TABLE 3:** Natural based nanoparticles used in ocular drug delivery.

Type of carriers	Drug/Carrier	Method of Preparation	Size (nm)	Entrapment Efficiency (%)	Advantages and Considerations	Ref
Lipoid nanoparticles (LNs)	Curcumin loaded in NLC Coated with chitosan-N-acethyl cysteine (NAC)	Melt-emulsification method	88.6	96.6	Topical administration in rabbit eye. Controlled release of drug for 72 h. Enhanced the retention time and corneal permeation with no toxicity and irritation	Li et al. (2016)
	Curcumin loaded in NLC	Hot melt emulsification/ultra-sonication method	66.8	96	Cur-NLC was stable for 3 months and could enhance the rabbit corneal permeability of Cur to 2.5 fold in comparison to Cur solution. So it is safe and effective formulation for anterior ocular drug delivery	Lakhani et al. (2018b)
	Quercetin loaded in Hybrid of NLC/hydrogel	Melt emulsification and ultra-sonication method	71–76	96.8–97.6	This PH and thermosensitive hydrogel system consists of CMCS and p407 that cross-linked by genipin (GP). Quercetin (QN) loaded into NLC/hydrogel to make QN-NLC-GEL-GP for ocular drug delivery that enhanced rabbit trans-corneal permeation and retention time thus improve the bioavailability of QN with no significant irritation. It could significantly increase the AUC of QN in comparison to the eye drop group (4.4 fold)	Yu et al. (2020)
	Quercetin in SLN	Melt emulsification method	143	66.5	Comparison of these two formulations, demonstrated that QT-SLN form shows better corneal permeability, more efficiency in protecting retina and corneal cells against stress oxidative, higher biocompatibility with corneal cells, and lower toxicity	Liu et al. (2015)
	Quercetin in Nanoemulsion (NE)		138.3	74.2		
	Baicalin in NLC based hydrogel (CMCS and F127 poloxamer cross-linked by genipin)	Melt emulsification-ultra-sonication method	99.6	89	This PH and thermosensitive hydrogel administrated as eye drops on rabbit eyes with no significant irritation and indicated a prolonged release profile. The corneal penetration enhanced in comparison with BN eye drops (4.46-fold)	Yu et al. (2019)
	Baicalin loaded in SLN	emulsification/ultrasonication method	91.4	62.4	Topical administration on the rabbit eye indicated no irritation. This formulation followed a prolonged release profile that enhance the bioavailability, corneal permeability, and stability of BN. It can be used for cataract treatment	Liu et al. (2011)
	Baicalein Lipid NPs (coated with trimethyl chitosan (TMC))	Thin film hydration method	162.8	90.6	To evaluate the trans-membrane permeability, the molecular dynamic stimulation was carried out. This topical formulation applied on rabbit eyes. The pre corneal retention time and ocular irritation indicated this formulation as a good carrier for ocular administration. The AUC of this formulation was increased 3.17 fold more than the control group and demonstrated sustained release profile. It used in the treatment and prevention of glaucoma and keratitis disorders	Li et al. (2020b)
	Tetrandrine in Cationic solid lipid NPs (TET-CNP)	Emulsion evaporation-solidification at low temperature	15.2	94.1	Topical administration on rabbit eyes indicated a prolonged drug release pattern with minimal toxicity in low concentration. Flow-cytometry results revealed more cellular uptake of TET-NP, so this formulation was more successful to apply in PCO.	Li et al. (2014)
	Tetrandrine in Anionic solid lipid NPs (TET-NP)	Emulsion evaporation-solidification at low temperature	18.7	95.6	Topical administration on rabbit eyes indicated a prolonged drug release pattern with minimal toxicity in low concentration. Flow-cytometry results revealed more cellular uptake of TET-NP, so this formulation was more successful to apply in PCO.	
	Tetrandrine in liquid crystalline nanoparticles (LCNPs)	Emulsion evaporation-solidification	1700	95.4	Topical administration of this formulation on rabbit eyes indicated more sustained release profile, corneal permeability, and enhanced bioavailability in comparison to the TET solution	Liu et al. (2016a)
	Genistein in NLC	Melt-emulsification technique followed by surface absorption of EDU RS 100	88.3	90.3	Topically instilled GEN-NLC applied in the rabbit eyes, the corneal permeation increased 3.3-fold in comparison to the NLC solution. The Draize test exhibited no irritation in cornea tissue. No significant toxicity in ocular tissues was reported. The AUC of this formulation was 1.22-fold more than bare NLC formulation. It would be a promising candidate for PCO treatment	Zhang et al. (2014a)
	Genistein in NLC	Melt emulsification technique	90.1	91.1	*In vitro* study showed this formulation could enhance GEN permeation into human lens epithelial cells (HLECs). It was effective in inhibiting the growth of (HLECs). The drug release profile demonstrated controlled and sustained release pattern in 72 h this formulation has the potency to prevent PCO.	Zhang et al. (2013)
	Genistein in NLC modified with Chitosan hydrochlorides	Melt emulsification technique combined with ultra-sonication	100–800	80.8–90.6	By decreasing the size of NPs, the cellular uptake into epithelial cells of the human lens increased that results in a promising carrier for PCO treatment	Zhang et al. (2014b)
	Genistein loaded in NLC	Melt emulsification technique	80.1	92.3	*In vitro* drug release demonstrated sustained drug release for 72 h. It would be useful for PCO prevention after cataract surgery	Liu et al. (2016c)
	Lutein in Nanoemulsion	Sonication method	10–12	NA	Sustained drug release in the first 24 h and release pattern reached a plateau at 144 h which enhanced the solubility and permeability of Lutein to the ocular tissues	Lim et al. (2016)
	Mangiferin NLC	Ultrasonication method	51.3	88.1	Topically administration of formulation indicated sustained drug release for 3 months and enhanced MGN ocular bioavailability, corneal permeability, retention time, and stability. The Draize test demonstrated good ocular tolerability and no ocular irritation	Liu et al. (2012)
	EGCG in Lipid nanoparticles CTAB (cationic lipid as a surfactant)	Double emulsion technique	90–300	98.9	These formulations enhanced the stability, safety, bioavailability, and biodegradability of EGCG for ocular drug delivery and for the treatment of ophthalmic disorders such as AMD, DR, and glaucoma through their anti-inflammatory and anti-oxidative effects. They also indicated a prolonged release profile with improved corneal resistance time. EGCG-DDABLNs shows 3-fold higher transscleral permeability than EGCG-CTAB LNs	Fangueiro et al. (2014)
	EGCG in Lipid nanoparticles DDAB (cationic lipid as a surfactant)	Double emulsion technique	130–380	96.8		
Micelle	Curcumin loaded in Nanomicelle with graft copolymer (PVCL-PVA-PEG)	Solvent evaporation/film hydration method	50.1	99.3	Topical administration in rabbit eye with no toxicity and irradiation. Enhanced the stability, corneal permeability, and anti-inflammatory effect of curcumin	Li et al. (2017)
	Curcumin in Micelle, (*in-situ* gelling system based PEG-DSPE/solutol HS 15 mixed with gellan gum	Solvent evaporation method	13.4	97.2	More permeability through the cornea than free Cur. The biocompatible *in-situ* gelling form increased the retention time on the rabbit cornea with good tolerability and no irritation	Sai et al. (2020)
	Curcumin loaded in Micelle	Solvent evaporation method	14–26	48–8	This eye drop formulation indicated the high potency of cur-micelle in reducing VEGF expression in retinal cells (D407) and protection of them against oxidative stress. It shows sustained release profile for 1 month that is suitable for treatment of chronic retina diseases such as wet and dry AMD.	Alshamrani et al. (2019)
	Curcumin in Micelle (ion sensitive *in-situ* gel used P123/TPGS mixed with gellan gum)	Thin film dispersion method	10.8	90.8	Topical administration of this *in-situ* gel system, show sustained release pattern with high biocompatibility, corneal permeation, and no irritation that makes it suitable for ocular drug delivery	Duan et al. (2015)
	Genistein loaded in Flt1 peptide–HA conjugate micelles	Sonication and dialysis method	172	40–50	Synergistic effect of GEN and Flt1 peptide in the anti-angiogenesis effect results in a beneficial treatment of ocular neovascularization. This inhibitory effect on vascular permeability and corneal neovascularization was demonstrated in diabetic retinopathy and silver-nitrate cauterized corneas of SD rats respectively. This formulation was enabled to control drug release for 24 h	Kim et al. (2012)
	Genistein in Micelle (MPEG-b-PAE-modified with HA)	Diafiltration method	84.5	NA	Topically instilled eye drops into the rabbit eyes. It was able to increase the retention time of formulation on the cornea thus enhance corneal permeation that leads to enhance the bioavailability of GEN. The anti-angiogenesis effect of this formulation makes it suitable for DR, CNV, and AMD treatment	Li et al. (2018)
	Resveratrol in Micelle	Film dispersion method	50.1	98.8	Topical instillation on rabbit eyes used for corneal wound healing. SOL-RES indicated no cytotoxicity, improved corneal permeability and cell proliferation, high ocular tolerance, more chemical stability, and good storage ability for 12 weeks	Li et al. (2020a)
Nanoparticles/Polymeric nanoparticles	Curcumin in B-cyclodextrin NPs modified with ethylene diamine (EDA)	Solvent evaporation method	189–300	NA	Topically drug delivery. Enhanced aqueous solubility and stability of Cur and improved corneal permeability	Liu et al. (2020)
	Curcumin in Albumin NPs (Thermoresponse *in-situ* gelling system) (Cur-BSA-NPs-Gel)	Desolvation method	221	85.4	Topical administration for the treatment of DR with sustained release profile and enhanced the bioavailability of Cur with no obvious irritation on rabbit eyes	Lou et al. (2014)
	Curcumin loaded in Albumin based Nanosphere	Desolvation method	203–354	NA	Albumin based nanospheres indicated promising efficiency in increasing the solubility and bioavailability, anti-oxidant property of curcumin, and they followed the sustained release pattern to release drug	Kim et al. (2019)
	Quercetin loaded in Chitosan NPs modified with PEG (co-delivery with Resveratrol)	Ionic gelation Using TPP as a cross-linker	308	81.3	Eye drop instillation reduced the IOP in normotensive rabbits. This formulation show Sustained drug release profile with enhanced the corneal permeability, and bioavailability of RES. The loading and EE% decreased by increasing the PEG concentration. PEG was used to modify CSNPs to reduce IOP in the glaucoma treatment. This formulation showed more radical oxygen (ROs) scavenging effects and corneal permeation than singular RES and CUR dispersion	Natesan et al. (2017)
	Resveratrol in PEG NPs Modified with chitosan	Ionic gelation method	129	91.8	RES-PEG-CS NPs administrated to the conjunctival cul-de-sac, demonstrated sustained release profile with sufficient corneal permeability to target the intraocular tissues with no irritation. It was efficient to reduce IOP and glaucoma treatment	Pandian et al. (2016)
	Naringenin loaded in sulfobutylether-β-cyclodextrin/chitosan nanoparticles	Ionic gelation method	446.4	67.1	The Draize test indicated no irritation on the rabbit eye. This formulation indicated the sustained drug release profile, enhanced bioavailability of NG with enhancing the solubility of NG and the retention time of the formulation on the surface of the eye, and reduced the frequency of drug administration	Zhang et al. (2016)
	Pilocarpine in PLGA NPs	Double emulsion method	82.7	57	This eye drops formulation administrated to the rabbit model. The ocular resistance time and ocular bioavailability increased in comparison to the commercial eye drop which results in more miotic response. Indicated initial burst release during the first 2 h, which continued by sustained release profile for up to 24 h	Nair et al. (2012)
	Pilocarpine in PCL Nanocapsules (NCs)	Double emulsion-solvent evaporation (Pluronic F68 as a surfactant)	235.4	89.2	Two types of nanoparticles (NCs, NSs) applied to rabbit eyes. NCs indicated better therapeutic efficiency in reducing IOP and sustained drug release for 42 days and higher entrapment and loading efficiency	Lee et al. (2017b)
	Pilocarpine in PCL Nanospheres (NSs)		227.7	30.1		
	Pilocarpine in Eudragit RL 100	Solvent displacement	121–291	41.6–72.9	Pilocarpine nanosuspension developed to enhance the drug availability, decrease the frequency of administration and sustained drug release for 24 h this formulation was safe and stable for ocular drug administration	Khan et al. (2013)
	EGCG in Gelatin nanoparticles decorated with HA (GEH NPs)	Self-assembly method	250	97	Topically administrated formulation on rabbit eyes (twice daily) was efficient in DES treatment. It was no toxic for HCECs cells, reduced the inflammation effect, prolonged the retention time on ocular surface, without making any irritation on the surface of the rabbit eye	Huang et al. (2018)
	EGCG in Gelatin Nanoparticles coated with RGD-HA	Self-assembly method	168.8	95	These NPs applied to the cornea of neovascularization mouse model. It was effectively target the specific receptor and significantly reduced the corneal neovascularization and prevented angiogenesis in cornea	Chang et al. (2017)
	Kaempferol in Gelatin NPs	Desolvation method	90	98	GNPs illustrated anti-angiogenic effect and reduced blood vessel formation on rat eyes so it can be used topically for the treatment of corneal neovascularization. Hematoxylin and eosin (H&E) stain, and metalloproteinases (MMP)/(VEGF) quantification demonstrated the efficiency of GNP-KA in reducing the number of corneal blood vessels	Chuang et al. (2019)
	Catechin in PEG NPs	Solvent evaporation method	5–200	NA	PEG/catechin formulation could enhance water solubility of catechin to 100-fold and exhibited a high anti-inflammatory effect that is ideal for the treatment of DES.	Shim et al. (2019)
Liposome	Thymoquinone and Latanoprost in Liposome	Thin film hydration method	99.4–150	88–92	(TQ) and (LAT) encapsulated in liposomal vesicles indicated the IOP lowering efficiency in glaucomatous rabbit eyes. Lip (LAT + TQ) and Lip (LAT) were the most effective formulations in lowering intraocular pressure for up to 48 h and enhancing sustained drug release without causing irritation on the eye surface. The drug loading efficiency for the liposomal form of TQ, LAT, and (TQ + LAT) reported 92%, 88%, and more that 88%, respectively. The particle size of these formulations increased by this order Lip (TQ) > Lip (LAT + TQ) > Lip (LAT) > Lip	Fahmy et al. (2018)
	Baicalin in Liposome/Transferosome penetration enhancer vesicles (PEVs)	Thin film hydration	667–1,341	41–99	Eye drops instillation on rabbit eyes, used for the treatment of cataract. These formulations (liposome, penetration enhancer vesicles PEVs, and transfersomes) indicated more anti-oxidative properties and bioavailability than BN solution. They demonstrated high encapsulation efficiency to sustain ocular drug delivery of baicalin for 3 months with no toxic effects and high ocular tolerability	Ashraf et al. (2018)
	Combretastatin in Liposome	Thin film dispersion method	109.2	74.3	The uptake efficiently of this formulation evaluated by human umbilical vein endothelial cells (HUVECs). This formulation could be useful in the treatment of chronic ocular disorders such as choroidal neovascularization (CNV) and DR.	Ma et al. (2009)
	Lutein in Liposome	Thin film hydration method	20-200	NA	It was beneficial in protecting the rabbits’ retina from DNA damage and the harmful effects of cisplatin. It also enhanced the efficiency of Lutein	Ibrahim et al. (2019)
	Berberine in Liposome	Thin film hydration method	103	89.6	PAMAM G3.0-coated liposomes improved corneal permeation and adhesion in the human and corneal epithelium of the rabbit. Moreover, enhanced berberine bioavailability and protective effect in human RPE cells and rat retina after photooxidative retinal injury. No toxicity and side effects were observed on rabbit ocular tissues. So it would be a promising formulation for ocular drug delivery and treatment of AMD disorder	Lai et al. (2019)
	BBR and CHR loaded in Liposome coated PAMAM G3.0	Thin film hydration method	148	93.9		
	Resveratrol in Nanogel (HCS and TPP as crosslinker)	Ionic gelation method	140	59	RES-HCS-NG used to controlled release of drugs. Efficient for treatment and prevention of ocular disease especially for AMD treatment	Buosi et al. (2020)
Nanogel	Pilocarpine in Nanogel (polyvinyl pyrrolidone/polyacrylic acid) (PVP/PAAc)	Ionized radiation method	80–120	NA	This formulation enhanced the stability, bioavailability, ocular retention time of pilocarpine. It showed sustained release profile for 24 h and reduced the frequency of administration. The loading efficiency was 12–48%	Abd El-Rehim et al. (2013)
	Curcumin entrapped in Nanogel in combination with cationic lipid nanoparticles (CNLC-GEL)	Film ultra-sonication technique	158.1	NA	The SOL-GEL transition temperature of Cur-CNLC-GEL was reported at 34°C. It follows the zero-ordered kinetics and increased 9.24 and 3.38 fold in AUC and Cmax of curcumin solution respectively. This formulation could enhance the bioavailability of Cur due to increase in corneal permeation and retention time	Liu et al. (2016b)
	Curcumin and latanoprost loaded in Chitosan- gelatin hydrogel	Emulsion-evaporation method	161.1	NA	Topical eye drops for glaucoma treatment. Sustained drug release for 7 days. Enhanced resistance time on rabbit eye and corneal permeation with minimal toxicity	Cheng et al. (2019)
Niosomes	Curcumin in Proniosomal gel	Coacervation phase separation method	212	96	Curcumin loaded in proniosomal gel indicated high biocompatibility, safety, and anti-inflammatory effects. This formulation increased the ocular retention time and corneal permeability. It showed sustained release profile over 24 h	Aboali et al. (2020)
Nanofiber	EGCG in SFNF	Electrospinning method	245	NA	EGCG-SFNF with anti-VEGF properties and controlled release pattern for 6 days can be considered as a promising scaffold for corneal tissue engineering and delivery system	Forouzideh et al. (2020)

### 3.1 Thymoquinone

Thymoquinone (2-isopropyl-5-methyl-1,4-benzoquinone) is the major biologically active ingredient of the volatile oil that is isolated from the medicinal plant *Nigella sativa* (Darakhshan et al., 2015). It has been demonstrated that this herbal ingredient shows neuroprotective, anti-inflammatory, and antioxidant effects (Alhebshi et al., 2013) and is effective in the treatment of glaucoma (Fahmy et al., 2018). Fahmy et al. (2018) reported various liposomal formulations of thymoquinone (TQ) and latanoprost (LAT) in reducing Intraocular pressure (IOP). Addition, this study indicated the promising role of TQ in the amelioration of retinal damage and the inflammatory responses in glaucomatous rabbits (Fahmy et al., 2018).

### 3.2 Catechin

Catechin (flavan-3-ol) is a member of the flavonoids, a class of natural polyphenols considered an antioxidant ingredient and found in various fruits, beverages, and tea. It has some biological advantages (Shimamura et al., 2007; Singh et al., 2011; Li et al., 2019). The therapeutic applications of catechin for ocular disease has been reported in various studies, including for dry eye, glaucoma, and various retinal disorders due to anti-inflammatory and anti-oxidative properties (Lee et al., 2017a). Lee et al. (2017a) investigated the nano-complex of PEG and catechin for enhancing the bioavailability and the therapeutical effect of catechin in the treatment of dry eye disease (DED). In another study Li and his coworkers employed a simple self-polymerization and self-assembly reaction to formulate a core-shell structure of polycatechin and gold nanoparticles (Au@Poly-CH NPs) as an eye drop to synergistically eliminate DED due to its antioxidant and anti-inflammatory effects (Li et al., 2019).

### 3.3 Epigallocatechin Gallate

Epigallocatechin gallate (EGCG) as a major ingredient of green tea, exhibits anti-inflammatory effects and is extensively used in the treatment of various inflammatory diseases and for the treatment of ocular disorders, such as AMD, DR, and DES (Fangueiro et al., 2016). However, the corneal epithelium is an effective barrier for hydrophilic EGCG. Luo and Lai (2017) designed new biodegradable gelatin-*g* poly (*N*-isopropyl acrylamide) (GN) nanocarriers for the topical administration of EGCG on rabbit eyes in the treatment of DED with anti-oxidant activity and sustained release profile (Luo and Lai, 2017).

### 3.4 Curcumin

Curcumin [1,7-bis(4-hydroxy-3-methoxyphenyl)-1,6-heptadiene-3,5-dione] is the yellow-colored bioactive component of turmeric powder, extracted from the rhizome of the plant *Curcuma longa* (Aggarwal et al., 2003) with a wide verity of physiological and pharmacological characteristics (Sharma et al., 2005; Shishodia et al., 2005; Sadeghi Ghadi and Ebrahimnejad, 2019; Sadeghi-Ghadi et al., 2020). Curcumin (CUR) is also considered as an effective ingredient for the treatment and prevention of various ocular disorders such as glaucoma, cataract, corneal and choroidal neovascularization, AMD, DR, DES (Radomska-Leśniewska et al., 2019). It can be beneficial in the treatment of proliferative epithelial disorders, the proliferation of human lens epithelial cells, and protects retinal cells, retinal ganglion cells, and corneal epithelial cells (Beevers and Huang, 2011). One of the major challenges with curcumin is low stability and storage difficulties. In order to overcome these problems, Maharjan et al. (2019) investigated different approaches to formulating curcumin or tetrahydrocurcumin (THC)-loaded in various derivatives of hydroxypropyl (HP)-cyclodextrins (CD) by applying the spray drying technique. It was reported that the stability, bioavailability and corneal and retinal epithelial permeability of curcumin (or THC) was significantly enhanced by encapsulating into the HP-CDs (Maharjan et al., 2019).

### 3.5 Berberine

Berberine (BBR), a type of isoquinoline alkaloid, is an active ingredient of *Rhizome Coptidis* and *Cortex Phellodendri* and extensively used in China for treating a variety of disorders (Lin et al., 2015; Choi, 2016; Wen et al., 2016). In order to improve the therapeutic effects, thermal stability of berberine and prevent it from oxidation, Lai and his coworkers investigated new liposomal formulations coated with G3 polyamidoamine dendrimer (PAMAM G3.0) for the treatment of AMD disease. They used berberine hydrochloride (BBH) and chrysophanol (CHR) in their formulations due to their anti-inflammatory and anti-angiogenesis effects for ocular drug delivery applications, respectively (Lai et al., 2019). According to previous studies, CHR can be beneficial in the treatment of retinal disorders due to its ability to suppress NF-κB/caspase-1 activation that leads to reduced inflammatory responses (Kim et al., 2010).

### 3.6 Pilocarpine

Pilocarpine, an alkaloid with an imidazole ring, is extracted from the leaves of the *Jaborandi* plant. It can be applied as a miotic agent for topical administration in glaucoma treatment. However, the corneal permeation of pilocarpine is restricted due to its high hydrophilicity that results in low ocular bioavailability. Nair et al. (2012) encapsulated pilocarpine in Poly (lactic-co-glycolic acid) (PLGA) nanoparticles to improve miotic effect and enhance the bioavailability and ocular retention time of pilocarpine.

### 3.7 Genistein

Genistein (4,5,7-trihydroxyisoflavone) is a flavonoid that’s abundant in soy products and has numerous pharmacological properties such as anti-oxidative, anti-inflammatory, and anti- angiogenesic (Aldina et al., 2019). It also considered a beneficial factor in the treatment and prevention of eye diseases, including DED, DR, AMD, cataract formation, and glaucoma (Lin et al., 2016). Genistein (GEN) can protect the cornea through its anti-inflammatory effect by suppression of oxidative stress (Xiao et al., 2018). It can also suppress IL-1β in the dry-eye model rat. Genistein can be used to prevent posterior capsular opacification (PCO) that’s the most common complication that occurs after cataract surgery due to the remained epithelial cells in the capsular bag that’s proliferated or migrated after cataract surgery which causes blurred vision (Spalton, 1999). So, genistein as an inhibitor of the growth of epithelial cells, can effectively reduce the frequency of PCO and enhance patient satisfaction after cataract surgery (Zhang et al., 2013).

### 3.8 Resveratrol

Resveratrol (trans-3,5,4′-trihydroxystilbene) is a non-flavonoid polyphenol compound that widely exists in dietary sources including grapes and peanuts. It has various therapeutic effects (Lee et al., 2011; Abengózar-Vela et al., 2019) and it also considered as a potential ingredient to prevent ocular disorders, including glaucoma, AMD, cataract, and DR (Bola et al., 2014) due to its free radical scavenging properties (Anisimova et al., 2011; Natesan et al., 2017). The small size and hydrophilic characteristics of resveratrol (RES) enables it to pass through the cornea and enter into the retina, however, the low bioavailability of RES restricts its applications. Several novel carriers have been explored for RES such as liposomes (Cote et al., 2015), β-cyclodextrin nanosponges (Abdallah et al., 2015), chitosan nanoparticles (Chuah et al., 2013), solid lipid nanoparticles (SLN) (Hippalgaonkar et al., 2013), protein complexes (Sahoo et al., 2011), poly-ε-caprolactone (PCL) (Wu et al., 2005), to overcome the low solubility, bioavailability, and stability issues of RES. Dong et al. (2019) synthesized gold nanoparticles (AuNPs) without utilising any harmful reductants. Resveratrol is used as a stabilizer and reducing agent in the fabrication of AuNPs. This formulation can reduce the permeability of the blood-retinal barrier in streptozotocin (STZ)-induced diabetic rats. According to the study’s findings, the number of retinal vessels and the expression of the vascular endothelial growth factor (VEGF-1) was decreased whilst the expression of renal pigment epithelium-derived factor (PEDF) increased in the retina of diabetic rats after administration of AuNPs (Dong et al., 2019).

### 3.9 Quercetin

Quercetin is the major prevalent flavonoid that is extensively found in various sources such as apples, tea, onions, nuts, berries, cauliflower, cabbage, and many seeds. Quercetin has many beneficial effects in the treatment and prevention of various diseases (Abengózar-Vela et al., 2019). It is beneficial in ocular disorders such as cataract, choroidal neovascularization (CNV), and AMD (Zhuang et al., 2011). It has antiangiogenic activity and a protective effect on human retinal pigment epithelium cells (Adelli et al., 2013). Subramanian et al. loaded RES and quercetin (QUR) in chitosan nanoparticles to reduce IOP in the glaucoma treatment (Natesan et al., 2017).

### 3.10 Naringenin

Naringenin [2,3-dihydro-5,7-dihydroxy-2-(4-hydroxyphenyl)-4H-1-benzopyran-4-one] is a flavonoid that belongs to the flavanones subgroup, is extensively found in several citrus fruits, figs, bergamot, and tomatoes. It has been shown to be useful in certain disorders (Salehi et al., 2019) including a beneficial effect on ocular disorders such as retinal pigment epithelium degeneration (RPE), choroidal neovascularization (CNV), and AMD and has attracted attention in recent years. Zhang et al. (2016) encapsulated Naringenin (NG) into sulfobutylether- β-cyclodextrin/chitosan nanoparticles (NG-CD/CS-NPs) for topical administration of the drug to treat AMD disorder.

### 3.11 Lutein

Lutein is a hydrophobic carotenoid with anti-oxidative and anti-inflammatory properties (Chang et al., 2018) and is found in green leafy vegetables, yellow fruits, petals of the marigold flower, orange, broccoli, spinach, kale, cilantro, corn, and egg yolk (Wallace et al., 2015). Lutein is found at high concentrations in macular pigment in the retina and functions as a light filter to protect the macula from UV-light and anti-oxidative damage. It has a protective effects in the treatment and prevention of ocular diseases (especially posterior eye diseases) such as DR, macular degeneration (MD), neuronal injury, AMD, uveitis, choroidal neovascularization, retinal ischemia, retinitis, and cataract (Koushan et al., 2013; Buscemi et al., 2018). Despite all these beneficial effects, the low stability, bioavailability, and solubility of lutein hinders its medicinal applications (Toragall et al., 2020). Liu et al. (2014) developed lipid nanoparticles and cyclodextrin for topical administration of lutein. This formulation was more successful in accumulating and partitioning lutein in the cornea, enhanced drug loading efficiency, stability, and decreased cytotoxicity than nanoparticles without lutein (Liu et al., 2014).

### 3.12 Kaempferol

Kaempferol [3,5,7- trihydroxy-2-(4-hydroxyphenyl)-4H-1-benzopyran-4-one] is a natural flavonoid that is found extensively in edible plants and fruits with high anti-oxidative, anti-inflammatory, anticancer, anti-angiogenesis, and antimicrobial activities (Hung et al., 2017; Du et al., 2018). In ocular disorders, it has attracted interest for the topical treatment of corneal neovascularization and its protective effect on RPE cells from reactive oxygen. Chuang et al. (2019) applied Kaempferol (KA) to hinder vessel formation and treat corneal neovascularization. To increase the bioavailability and sustained release of KA, it was loaded into gelatin nanoparticles (GNP) for administration as eye drops for ocular drug delivery (Chuang et al., 2019).

### 3.13 Baicalin

Baicalin (5,6-dihydroxy7-O-glucuronide) is a flavonoid with low water solubility and stability and is extracted from the *Scutellaria baicalensis Georgi* plant (Ashraf et al., 2018). Baicalein, baicalin, and wogonin are among the major bioflavonoids extracted from it. They can be used for the treatment of various diseases (Natesan et al., 2017; Sowndhararajan et al., 2018). They exhibit a broad spectrum of biological activities in the eyes, such as anti-inflammatory, antibacterial, anti-cataract, antioxidant, and anti-angiogenesis effects and can be effective in the treatment of AMD, DR, and uveitis (Natesan et al., 2017).

Ashraf et al. (2018) formulated three different nanostructural systems to enhance baicalin (BN) pharmacological and physiological properties (Liu et al., 2009). In other studies, the efficiency of SLN and NLC for the delivery of baicalin for the treatment of cataractic rats was investigated (Liu et al., 2011).

### 3.14 Tetrandrine

Tetrandrine (6,6′,7,12-tetra methoxy-2,2′-dimethyl-1 beta-berbamane) is an alkaloid extracted from the Chinese medicinal herb *Radix Stephania tetrandrae S* with anti-inflammatory, immunologic and antiallergenic effects. It has beneficial effects on ocular disorders and can be used in the treatment of opacification of the posterior lens capsule, cataracts, glaucoma, chronic keratitis, retinopathy, and ocular inflammations (Huang et al., 2011). Li et al. loaded tetrandrine in cationic solid lipid nanoparticles (TET-CNP) and anionic solid lipid nanoparticles (TET-NP) to enhance the bioavailability of TET (Liu et al., 2016a). They demonstrated that negatively charged NPs are more efficiently uptaken into the cellular human lens compared to the cationic TET-CNP, thus the formulation could be effective in PCO treatment (Li et al., 2014).

## 4 Application of Nanotechnology for Ocular Drug Delivery

Nanotechnology has been extensively explored in the medical field in recent years, in both the diagnosis and treatment of diseases (Mir and Ebrahimnejad, 2014; Jafari et al., 2016). The advent of nanotechnology promises to accelerate improvements in ophthalmologic drug delivery systems (Kamaleddin, 2017). These novel drug delivery systems aim to facilitate the efficient permeation of drugs through complex ocular barriers (Liu et al., 2016b; Cheng et al., 2019), thus enhancing the therapeutic effect (Lee et al., 2017a; Lee et al., 2017b) and bioavailability (Lee et al., 2017a; Lee et al., 2017b) compared to conventional drug delivery systems. Nanoparticles (NPs) can be designed to prevent drugs from degradation (Beloqui et al., 2016; Lee et al., 2017b), improve penetration through ocular barriers (Priwitaningrum et al., 2016), drug targeting (Hornung et al., 2015), and sustain drug release (Hornung et al., 2015; Yang et al., 2016) and thus enhance efficacy.

Nanotechnology introduces many novel nanocarriers for the treatment of ophthalmic disorders by modification and formulation of existing drugs that lead to an increase in the number of commercial nano-based drugs, in the ocular drug delivery area. Despite much progress in this field, there are few FDA-approved nanomedicine drugs in the market (Table 4), and many of them are in their early stage of clinical development (Reimondez-Troitiño et al., 2015).

**TABLE 4:** FDA approved nanomedicine applied in ocular diseases.

Product	Formulation	Active Ingredient	Indication	Route of Administration	Date of Production	Ref
Restasis^®^	Nanoemulsion	Ciclosporin A	Chronic Dry eye	Eye drop	1983	Reimondez-Troitiño et al. (2015)
Visudyne^®^	Liposome	Verteporfin	AMD	Intravitreal injection	2000	Pooja et al. (2018)
Macugen^®^	Aptamer–polymer nanoparticle	Pegaptanib sodium	Neovascular (wet) AMD	Intravitreal injection	2004	Pooja et al. (2018)
Retisert	Non-biodegradable implant	Fluocinolone acetonide	Uveitis	Intravitreal implant	2005	Reimondez-Troitiño et al. (2015)
Triesence	Suspension	Triamcinolone acetonide	Macular edema	Intravitreal or periocular injection	2007	Khiev et al. (2021)
Durezol^®^	Nanoemulsion	Difluprednate	Eye inflammation	Eye drop	2008	Reimondez-Troitiño et al. (2015)
Trivaris	suspension	triamcinolone acetonide	uveitis	intravitreal Injection	2008	
Ozurdex	Biodegradable implant	dexamethasone	Uveitis/diabetic macular edema	intravitreal implant	2009	Khiev et al. (2021)
Kenalog	Suspension	Triamcinolone acetonide	Macular edema	Intravitreal Injection	2009	
Iluvien	Non-biodegradable implant	Fluocinolone acetonide	Diabetic macular edema	Intravitreal implant	2014	

**FIGURE 4:**
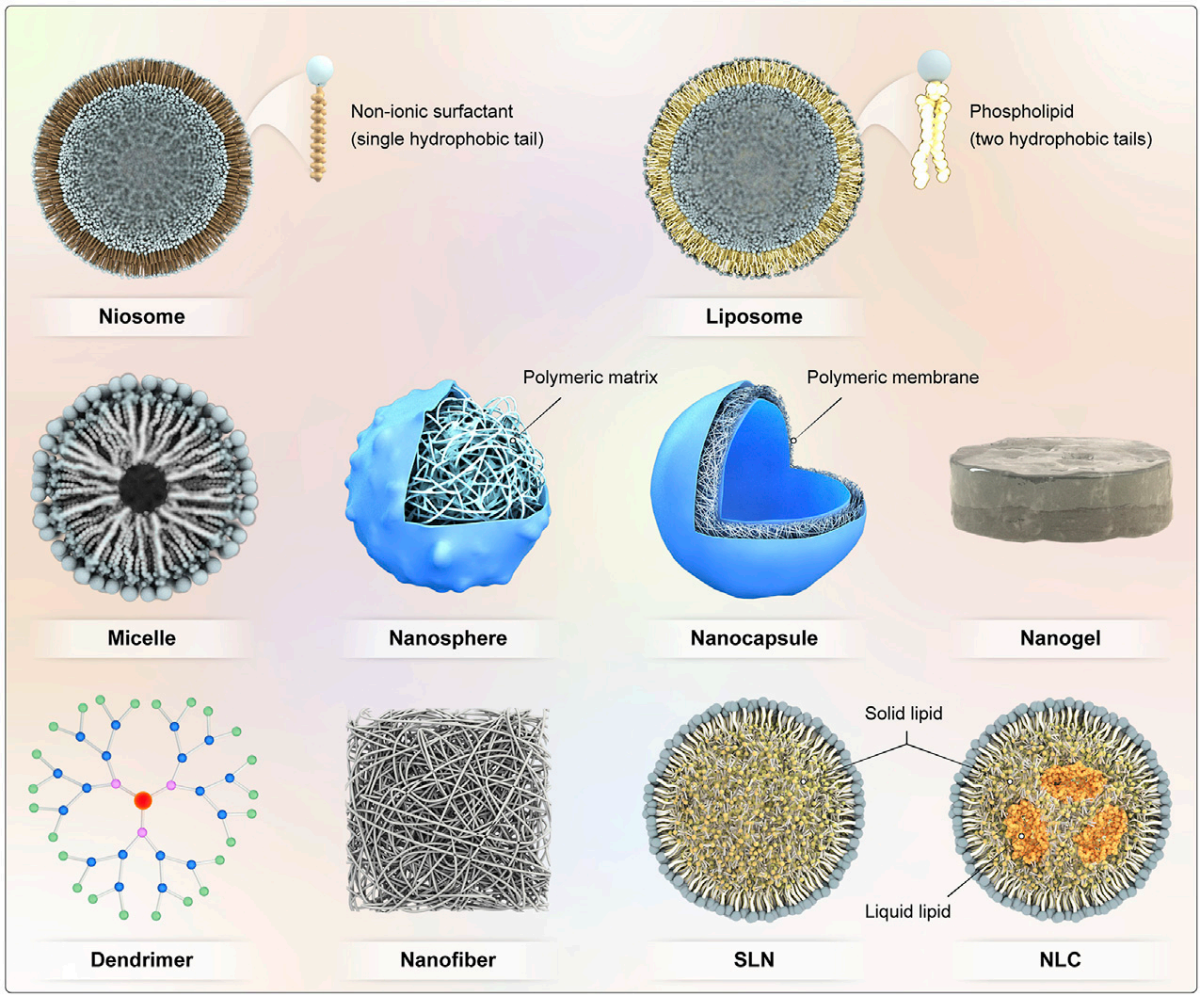
Various nanocarriers for ocular drug delivery.

In ocular drug delivery, the ability of NPs to adhere to an ocular tissue, mucosa, and epithelium is a major benefit and prevents the formulations from being washed away immediately by ocular defense mechanisms (Yu et al., 2020; Sai et al., 2020). Various types of nanotechnology have been investigated to improve the ocular drug delivery (Omerović and Vranić, 2019). Nanostructured carriers have emerged as minimally invasive drug delivery systems (Zhang et al., 2016; Wu et al., 2011), which can preserve therapeutic drug concentrations in the eye for extended times (Shen et al., 2015; Zhang et al., 2016), reducing the need for frequent administration (Battaglia et al., 2016), and reducing the side effects (Lai et al., 2019; Sultana et al., 2011). To date, various nanocarriers such as polymeric NPs, lipid NPs, liposomes, niosomes, micelles, dendrimers, and nanofibers (Figure 4) have emerged as novel technologies to overcome ocular barriers and improve drug delivery of therapeutic drugs to target sites with enhanced ocular bioavailability (Hironaka et al., 2009; Madni et al., 2017). The small size and adjustable physicochemical and functional properties provide advantages for delivering drugs to target sites (Patra et al., 2018). In this review we provide an overview of nanoparticles and nanofibers that have been explored for the ocular drug delivery of natural products. The various nanocarriers used in ocular drug delivery applications in the treatment of glaucoma, corneal diseases, corneal neovascularization, choroidal neovascularization, age-related macular degeneration, will be considered.

### 4.1 Polymeric Nanoparticles

Polymeric nanoparticles (PNPs) are carriers composed of biodegradable and biocompatible natural or synthetic polymers, with or without mucoadhesive properties. Both synthetic polymers such as polyacrylamide, polyacrylate, PCL, PEG, and PLGA (Ebrahimnejad et al., 2009; Wilczewska et al., 2012) and natural polymers such as gelatin, albumin, DNA, sodium alginate, carboxymethylcellulose sodium (CMC), and chitosan can be used to produce PNPs (Varshochian et al., 2015; Madni et al., 2017). They can deliver drugs from either active ingredients adsorbed on the surface or by having it encapsulated into the particle itself. Nanoparticles can be classified as nanospheres (NSs) and nanocapsules (NCs). NSs represent a matrix delivery system where a drug is adsorbed on the surface of the matrix or dispersed within it (Khalili and Ebrahimnezhad, 2017; Omerović and Vranić, 2019). NCs are vesicular systems where the inner core has different properties to the outer polymeric layer and they consist of film polymeric cover wrapping around an oil-filled chamber with a size distribution typically in the range from 10 to 1,000 nm (Paolicelli et al., 2010). In these systems, a drug is commonly dispersed in the core of the particle, but it may also be adsorbed on the surface. The drug loading efficiency is dependent on the affinity between drug and polymers, and the number of functional groups in the polymers for interaction with drugs. In one study, Lee and his coworkers synthesized two types of nanoparticles for long-term and prolonged release of pilocarpine in glaucoma therapy. They used poly (*ε*-caprolactone) to prepare nanocapsules and nanospheres harboring or encapsulating drugs for ocular drug delivery. It was demonstrated that the loading efficiency of pilocarpine in the PCL NCs was significantly higher than that the PCL NSs and drug release followed a sustained release pattern. The bioavailability, degradation rate, and *in vivo* experiments on rabbit eyes indicated that PCL NCs are a promising carrier for the treatment of glaucoma, and most effectively reduced the intraocular pressure of rabbit eyes (Lee et al., 2017b). Ruginǎ et al. (2019) loaded RES as an anti-VEGF agent in micro/nanocapsules [composed of polyelectrolytes coated with rhodamine 6G (Rh6G)] to deliver RES into retina pigmented epithelial D407 cells to treat diabetic retinopathy. In another study, Kim et al. (2019) formulated nanospheres with bovine serum albumin and evaluated the potency of antioxidant protection of rosmarinic, ursolic acid, and curcumin in the retina epithelial cells. It was demonstrated that these formulations increased drug solubility and bioavailability and decreased the production of ROS in retina tissues, thus albumin nanospheres could be a promising carrier to deliver anti-oxidative drugs to the anterior and posterior chamber of the eye. There are a number of approaches that can be considered to enhance the absorption of nanoparticles by increasing the retention time such as the use of mucoadhesive polymers, and optimizing nanoparticle size (Ebrahimnejad,, 2009; Ebrahimnejad et al., 2011; Tahara et al., 2017; Sharifi et al., 2019). Bodoki et al. (2019) developed novel nano-formulation by using zein and PLGA nanoparticles to form nano-gels with mucoadhesive and thermosensitive properties for topical administration to enhance the bioavailability, stability, and retention time of lutein for ocular drug delivery. The efficiency of these formulations was evaluated on the selenite-induced rat model of cataracts. The obtained results demonstrated that topically applied lutein-NPs significantly reduced the cataract intensity in comparison to free ocular lutein and oral delivery (Bodoki et al., 2019). Recently, polymeric nanoparticles in the size range from 10–200 nm have gained considerable attention as carriers for ocular drug delivery (Sabzevari et al., 2013; Badiee et al., 2018), due to their ability to enhance bioavailability (Ogunjimi et al., 2017). This indicates that increasing the size of functionalized nanoparticles decreases bioavailability. Therefore, to target the posterior segment of the eye the functionalized nanoparticles size should be kept around 200 nm. The physicochemical properties of nanoparticles enhanced absorption and penetration to the retinal glial cells. Nanoparticle charge is an important parameter, for example changing the negative charge particle to become cationic resulted in NPs penetrating deeper into ocular tissues (Madni et al., 2017; Bisht et al., 2018).

PEGylation is one of the most frequently used approaches to modify the surface of carriers in order to influence the permeability, retention time, and absorption of drugs. Pandian et al. (2016) applied PEG to surface modify chitosan nanoparticles. Resveratrol (RES) was used as a drug model for glaucoma treatment and loaded into nanoparticles. The results indicated an excellent correlation between an increase in PEG concentration and the size and polydispersity index of formulated nanoparticles. The release profile of RES indicated an initial burst release that was followed by a sustained release profile. The irritation test of formulations was evaluated by Hen’s Egg test and results demonstrated the safety of these formulations for ocular drug delivery. These surface modified formulations significantly reduced the IOP within rabbit eyes and enhanced drug permeation through the cornea (Pandian et al., 2016). Chang et al. (2017) developed gelatin/epigalloccatechin-3-gallate nanoparticles coated with conjugated complex comprised of an arginine–glycine–aspartic acid (RGD) peptide grafted to hyaluronic acid (HA) to target α_v_β_3_ integrin on human umbilical vein endothelial cells (HUVECs) for treatment of corneal neovascularization of mouse eye. Surface plasmon resonance was used to confirm to the binding of NPs to the integrin α_v_β_3_. The drug release demonstrated a sustained release profile. These nanoparticles are considered a promising carrier to inhibit the vascular endothelial cells and target the specific site of action (Chang et al., 2017).

### 4.2 Micelles

Micelles are composed of monolayers of amphiphilic agents (e.g., lipids, polymers) that can self-assemble in aqueous media. The particle size of micelles range between 10 and 100 nm. Micelles show more ordered structures than liposomes but exhibit various structures that depend on the hydrophobic and hydrophilic properties of molecules and solvents. The concentration of polymers in solution is a determining factor in the formation of micelles thus the critical micelles concentration should be attained in order to obtain core-shell nanocarriers with a hydrophobic core and a hydrophilic shell (Cagel et al., 2017). Hydrophobic drugs and active ingredients can be encapsulated and protected in the hydrophobic core of micelles in order to deliver them to the target site and enhance permeation of drugs through the epithelial layers which leads to reduced side effects and increased bioavailability. The hydrophobic shell can be utilized to control release and also specific targeting by immobilizing targeting moieties on the surface of micelle. Li et al. (2017) formulated curcumin nanomicelles as a topical ophthalmic formulation decorated with polyvinyl caprolactam-polyvinyl acetate-polyethylene glycol (PVCL-PVA-PEG) as a graft copolymer. This functionalized nanomicelles improved the solubility, stability, encapsulation efficiency, antioxidant properties of curcumin, and was well tolerated in rabbit eyes. Moreover, it enhanced the corneal permeation and anti-inflammatory properties of curcumin, indicating it as a promising carrier in ophthalmology (Li et al., 2017). Additionally, nanomicelles demonstrate a low critical micelles concentration, stability in solution, a high solubilization capacity, and low cytotoxicity (Mandal et al., 2017). The mucoadhesive nature and small size of polymeric micelles were evaluated and the results from both *in vitro* and *in vivo* animal studies indicate that polymeric micelles can enhance contact time with the ocular surface and improve drug transport through intraocular tissues *via* the paracellular route (Suri et al., 2020). Moreover, the hydrophilic nature of polymeric micelles produces clear solutions that can be used in the form of eye drops without any visual disturbance. Polymeric micelles can therefore be considered as one of the most promising techniques in ocular drug delivery for the treatment of both anterior and posterior segment of eye diseases such as DES, AMD, DR, glaucoma (Alviset et al., 2022), endophthalmitis, retinitis and corneal or conjunctival squamous cell carcinoma (Madni et al., 2017). Li et al. (2020a) evaluated a Soluplus micelle of resveratrol (SOL-RES) for corneal wound healing. In a separate study of cellular uptake and corneal permeation, coumarin-6 loaded within nanomicelles. The irritation test and histopathological observation of rabbit corneas were evaluated 24 h after eye drops instillation and results indicated good ocular tolerance and no eye irritation (Li et al., 2020a).

### 4.3 Nanofibers

Nanofibers are fibers with diameters in the range of 1–100 nm. They provide a large surface area up to 1,000 m^2^ per Gram that can enhance drug loading capacity (Deepak et al., 2018). Various natural (e.g., chitosan, fibronectin, gelatin, collagen, silk, and ethylcellulose) or synthetic polymers (e.g., PLA, PGA, PLGA, PEO, PCL, and PVA), or combinations can be used to produce nanofibers through the electrospinning process. Nanofibers can be modified by varying parameters such as the concentration of polymer solution and drug, adjusting porosity (Goyal et al., 2016), morphology and the diameter of fibers. Moreover, they can be functionalized to modulate the drug release (Zong et al., 2022). They can provide sustained-release profile that results in a reduction in the frequency of administration and thus enhance patient compliance (Gelb et al., 2022). Attractive physical properties of nanofibers such as the high surface-area-to-volume ratio, high porosity, flexibility, high drug-loading capacity, biocompatibility, biodegradability and increasing the contact time of drug with target tissues make them a unique candidate for drug delivery applications, diagnosis and treatment of different diseases, particularly for chronic ocular diseases that require frequent drug administration. Moreover, they can provide a surface for growth, attachment, differentiation, and proliferation of cells (Goyal et al., 2016). Forouzideh et al. (2020) investigated the beneficial anti-angiogenesis effect of silk fibroin nanofibers (SFNF) loaded EGCG on corneal tissue. The nanofibers prepared by electrospinning technique were characterized, the drug release studies of nanofibers showed a controlled release pattern over 144 h, and drug loading of EGCG into the silk fibroin nanofiber reported at approximately 8.0%. MTT assay and human umbilical vein endothelial cells (HUVEC) were used to determine the toxicity and appropriate dose of the drug. Results demonstrated EGCG in nanofiber lead to inhibition of HUVEC and provide an appropriate environment for hosting and proliferation of limbal cells. Moreover, SFNFs with a rough surface provide good conditions for attachment and adhesion of cells on the surface of nanofiber that makes it a promising scaffold for corneal tissue engineering (Forouzideh et al., 2020).

### 4.4 Dendrimers

Dendrimers are nano-sized, three-dimensional, hyperbranched, and typically star-shaped structures with many arms emerged symmetrically from a central core (Patri et al., 2002). The size of these structures is related to the various generations (G0, G1, and G2, etc.). Dendrimer nanoparticles can be produced by fast reduction and nucleation reactions (Crooks et al., 2001). The size of dendrimers is usually smaller than 100 nm. The synthetic dendrimers most commonly used in nanomedicine include polyamidoamines (PAMAM), poly (l-lysine) (PLL), polyesters (PGLSA-OH), polypropylimines (PPI), poly (2,2-bis (hydroxymethyl)propionic acid), and aminobis (methylenephosphonic acid) (Mignani et al., 2013). Hydrophobic drugs can be encapsulated in the core or entrapped among the branches of dendrimers based on the properties of polymers used in their construction. The surface of dendrimers can be modified by attaching molecules that may result in increasing the interaction of the dendrimer with biological membranes and high drug payloads. The small size, multi-functional properties, high drug loading ability, water-solubility, targeting ability by surface modification, bioavailability, and biocompatibility make dendrimers a promising candidate for drug delivery (Yavuz et al., 2015; Rodríguez Villanueva et al., 2016). Moreover, their low polydispersity index prevents them from uptake by the reticuloendothelial systems, thus enhancing drug permeation. PAMAM dendrimer have been the main family of dendrimers investigated for drug delivery (Chaplot and Rupenthal, 2014). Yang et al. (2012) indicated that the bioavailability of anti-glaucoma drugs in the cornea of rabbits was enhanced and intraocular pressure decreased by using a hybrid of PAMAM dendrimer hydrogel/PLGA formulation (Yang et al., 2012). PAMAM dendrimer could be used to reduce the frequency of topical ocular administration. The influence of size, molecular weight and various type of surface groups in poly PAMAM dendrimers was investigated in a controlled ocular drug delivery by Vandamme et al. Pilocarpine and tropicamide were loaded in different dendrimer formulations to evaluate the miotic and mydriatic activities, the tolerability, and residence time of dendrimer solutions on the ocular surface of rabbits. The obtained results indicated that the retention time of dendrimers with carboxylic and hydroxyl surface groups was longer than the other formulations. However, altering dendrimer concentration had no significant effect. Moreover, this study demonstrated the influence of size, molecular weight, charge, and geometry of dendrimers on ocular residence time (Vandamme and Brobeck, 2005).

### 4.5 Lipid Nanoparticles

Lipid nanoparticles (LNPs) can be considered as oil/water (o/w) emulsions where liquid lipids are replaced with solid lipids at room temperature. LNPs can provide prolonged drug release with negligible toxicity, so they may be explored as promising carriers for ocular therapeutics. LNPs are classified into two groups: solid lipid nanoparticles (SLNs) and nanostructured lipid carriers (NLCs) (Omerović and Vranić, 2019). SLNs are colloidal lipid-based systems with an average diameter from 50 to 1,000 nm (Müller et al., 2000) and composed of high melting point lipids, water, surfactants, and cosurfactants that stabilize the liquid dispersion (Lingayat et al., 2017). A broad spectrum of lipids can be used to produce SLN include triglycerides, partial glycerides, fatty acids, steroids and waxes (Mehnert and Mäder, 2012; Dudhipala, 2019). Different methods can be applied to produce SLNs such as hot homogenization methods, micro-emulsion method, coacervation method, solvent evaporation, and solvent diffusion from emulsions, solvent injection method, ultrasonication, supercritical fluid extraction of emulsions, and precipitation method (Mukherjee et al., 2009; Silva et al., 2011; Battaglia et al., 2014; Naseri et al., 2015; Rajpoot, 2019). SLNs can be used for different routes of drug administration such as oral, rectal, topical, ophthalmic, parenteral, and other routes (Azhar Shekoufeh Bahari and Hamishehkar, 2016; Beloqui et al., 2016; Bhagurkar et al., 2017). SLNs have the ability to entrap hydrophilic and hydrophobic drugs, are physically stable, prevent the degradation of encapsulated drug, enhance drug bioavailability and biocompatibility (based on the kind of lipids used), and a production process that is simple and cost-effective (and without requiring organic solvents), and the ability to be sterilized and produced at an industrial scale (Beloqui et al., 2016). The biocompatibility and mucoadhesive properties of SLNs, cause to enhance their interaction with the eye mucosa and drug retention time on the eye surface and let it pass the corneal barrier. The negatively charged epithelium provides an opportunity for cationic SLN particles to enhance the drug retention time on the eye and increase its absorption (Bonilla et al., 2022). Despite the numerous advantages, SLNs suffer from numerous disadvantages such as limited drug loading due to the solid crystalline state of the nanoparticles and burst release of both hydrophilic and hydrophobic drugs to the solubility of the drug in the lipid melt (especially remarkable in hydrophilic drugs *via* adsorption to the surface of SLNs and in polar drugs *via* existence in outer surfactant layer). Thus, the second generation of lipid nanoparticles introduced to eliminate these drawbacks were NLCs that composed of a mixture of solid and liquid lipids. Different kinds of NLCs can be prepared by applying various concentrations of liquid lipids and different methods of production. Utilizing the liquid lipid in the NLCs leads to enhanced drug loading, increased drug solubility, and reduces the crystallization of solid lipid that minimises the burst release of drug (Tian et al., 2012). NLCs have been extensively applied for anterior and posterior segment ocular drug delivery *via* corneal and non-corneal pathways (Tian et al., 2013; Zahir‐Jouzdani et al., 2019). The size and surface charge of lipid nanoparticles have an important role in the potential targeting and the extent of drug permeation, in this approach reducing the size of LNPs increases trans-corneal absorption (Kalam et al., 2010) and a positive charge results in higher permeation than a neutral or negative charge and enhances the retention time of nanoparticles on the surface of the cornea (Tamilvanan and Kumar, 2011), however, cationic particles may cause irritation and have toxic effects on ocular tissue due to a greater electrostatic interaction with the anionic layer of ocular tissue thus non-ionic surfactants and lipids preferred (Naseri et al., 2015; Üstündağ Okur et al., 2015). Fangueiro et al. (2016) evaluated *in vivo*, *ex vivo*, and *in vitro* studies on EGCG loaded cationic lipid nanoparticles (LNPs) produced by the double-emulsion technique. The pharmacokinetic profile of the corneal permeation of EGCG loaded into two different formulations of LNPs were evaluated and obtained results of EGCG cetyltrimethylammonium bromide (CTAB) LNs and EGCG-dimethyl dioctadecyl ammonium bromide (DDAB) showed a Boltzmann sigmoidal profile and first-order kinetics respectively. They utilised natural lipid in the formulations that are considered safe, biocompatible, and biodegradable. These cationic lipids indicated high stability without making no irritation or any toxic effects. The positive charge of these LNPs can interact with negative charge of mucosa on the surface of the eye which leads to higher retention time and enhanced permeation through trans-scleral and trans-corneal pathways (Fangueiro et al., 2016; Bodoki et al., 2019). Yu et al. formulated a hybrid pH and thermo-sensitive hydrogel of NLCs for ocular delivery of quercetin. Carboxymethyl chitosan (CMC), and poloxamer 407 were used in hydrogel construction and genipin (GN) used as a crosslinker. Fluorescence imaging, confocal laser scanning microscopy (CLSM), and *ex-vivo* transcorneal experiments demonstrated that NLCs enhanced corneal permeability and retention time. To evaluate the cellular uptake an *ex-vivo* transcorneal study was undertaken. Coumarin 6 was used as a hydrophobic fluorescence marker that was administrated into rabbit eyes. Intraocular permeation and distribution of Coumarin 6 were imaged by CLSM after 30 and 120 min of drug instillation. According to the findings, the corneal retention time followed an order of: NLC-Gel > Gel > NLC > eye drops. Cytotoxicity tests and histological examination demonstrated the safety and cytocompatibility of the NLC-Gel formulation (Yu et al., 2018; Yu et al., 2019; Yu et al., 2020).

### 4.6 Liposomes

Liposomes are spherical vesicles with phospholipid bilayers surrounding an aqueous core. The encapsulated drug in these systems can be protected by the lipid bilayer that leads to controlled drug release (Fakhravar et al., 2016). Phosphatidylcholine, cholesterol, and lipid-conjugated hydrophilic polymers are among the common components found in their structures. The size of liposomes ranges from 25–2,500 nm (Akbarzadeh et al., 2013). Liposomes are biodegradable, biocompatible, and nontoxic carriers that can encapsulate both hydrophilic and hydrophobic drug molecules. Despite these superior properties, liposomes suffer from instability due to the presence of unsaturated lipids in their structures that may be hydrolyzed or oxidized and causes the leakage of encapsulated drug. Moreover, aggregation and fusion of liposomes prevent them from ocular tissue absorption. To overcome this limitation, positively charged liposomes were introduced to increase corneal absorption and resistance time. There are numerous methods for producing liposomes which include: thin-film hydration (Zhang, 2017; Zhao et al., 2017), size reduction sonication, reverse-phase evaporation (Shi and Qi, 2018), solvent injection (Sharma et al., 2018), detergent depletion (Salimi, 2018), supercritical fluid process (William et al., 2020), high-pressure homogenization (Ibišević et al., 2019), and low-pressure extrusion (Rameez et al., 2010). Liposomes can be considered as a good carrier for sustained and triggered drug release (Oude Blenke et al., 2013) and have the potential to use for ocular drug delivery (Vafaei et al., 2015; Goyal et al., 2016). They are able to increase the contact time within ocular tissues, thus improve drug absorption and enhance ocular bioavailability and also patient satisfaction due to reducing the dosing frequency. In liposomal systems, drugs can be protected against enzymatic degradation in tear film or/and corneal epithelium which results in a reduction in the clearance rate of the formulations (López-Cano et al., 2021). Mucoadhesive and permeation properties of liposomes can be enhanced by surface modification. Surface charge and size of liposomes have a great effect on ocular drug delivery and the degree of drug permeation into ocular tissues. The critical role of size and charge of liposome on corneal permeation was considered by Schaeffer and Krohn where they applied the formulations in rabbit models. They demonstrated that the permeation of topically administrated formulation through the cornea of rabbits increased in the order small cationic unilamellar vesicles (SUV+) *>* multilamellar anionic vesicles (MLV-) *>* small anionic unilamellar vesicles (SUV-) *>* SUV *>* MLV free drug (Lakhani et al., 2018a; Venkatraman et al., 2018). In another study, Hironaka et al. (2009) indicated that liposomes smaller than 200 nm show better absorption into retinal tissue whilst particles larger than 600 nm exhibit minimal absorption. According to the obtained results, liposomes can enhance the pharmacokinetic profile of drugs so they can be applied for the treatment of the anterior and posterior segment of the ocular diseases such as glaucoma, DME, ARD, DR, endophthalmitis, retinitis, and corneal or conjunctival squamous cell carcinoma (Kompella et al., 2013). However, the applicability of liposomes has been hindered by issues such as low stability and poor reproducibility, low encapsulation efficiency, uptake by the reticuloendothelial system during phagocytosis, and cause visual cloudiness when intravitreal injected. ER et al. (2021) loaded curcumin and rhodamine B (RhB) dye into multilamellar liposome (MLV) by the thin-film hydration method. They used sodium alginate (SA) and acrylic acid (AA) grafted to each other through a radical polymerization method. Since riboflavin (RB) works as a transporter through the blood retina barrier (BBB), it was conjugated to the produced SA-g-AA to facilitate efficient delivery into the retina region. The resultant product (SA-g-AA-RB) was coated on the surface of the produced MLV using the o/w emulsion method that followed by ionotropic gelation to construct MLV-SA-g-AA-RB carriers that are able to target the retinal region. This formulation produced a prolonged-release profile, good membrane permeability, cellular absorption, and good bioavailability. The size of MLV-SA-g-AA-RB was reported at 730.5 nm while the size of MLV-SA-g-AA with CUR and RhB was 981.7 nm. The results demonstrated that the small size of liposomes produce higher uptake by cells. The encapsulation efficiency (EE) of CUR and RhB was 61 and 66%, respectively, and both showed a controlled manner of drug release. These formulations can be considered as an appropriate carrier to target and deliver drugs to the retina tissue (ER et al., 2021). In another study, Ibrahim et al. (2019) evaluated the protective and anti-oxidative effects of liposomal forms of lutein in cisplatin-induced retinal injury in rabbit eyes. Liposome prepared by the thin-film hydration technique were injected into the peritoneal cavity. Intraperitoneal injections were repeated twice per week for 2 weeks. The rabbit retina was analyzed by the Comet assay, electroretinogram (ERG), and histopathological examination. The result demonstrated that liposomal lutein formulation could be beneficial to avoid the deleterious effects of cisplatin on the rabbits’ retina and prevent DNA and histopathological damage (Ibrahim et al., 2019).

### 4.7 Niosomes

Niosomes are formed by the self-assembly of non-ionic surfactants that form closed bilayer vesicles in aqueous media. They are biocompatible and biodegradable in nature. They are able to entrap both hydrophilic and hydrophobic drugs (Sadeghi Ghadi et al., 2019). They have enhanced chemical stability, mechanical rigidity, safety, bioavailability, and entrapment efficiency compared to liposomes (Gan et al., 2013). However, hydrolyzation and leakage of the drug are the major disadvantages of niosmes. To improve the stability of niosomes against enzymatic degradation, cholesterol can be used in their formulations. Solulan, chitosan, carbopol, and dicetylphosphate are among the non-ionic surfactants that are utilized for ocular formulations (Wadhwa et al., 2009). Niosomes are considered a promising system for topical drug delivery to the eye for the treatment of ocular disorders due to controlled drug release, ability to deliver drug to the target site with no ocular irritation or side effects, and enhanced bioavailability (Sultana et al., 2011). Jain et al. (2020) developed a niosomal gel formulation of pilocarpine instilled into the lower conjunctival sac of rabbit eyes for glaucoma treatment. Pilocarpine niosomes prepared by the ether injection technique, a nonionic surfactant such as Span 20, 60, 80, and various molar ratios of cholesterol evaluated to optimize the niosomal formulation. The optimized formulation integrated into Carbopol 934 and locust bean gum-based gels. Pilocarpine niosomal gel formulation enhances the bioavailability, stability, and precorneal retention time of niosomes. This study showed that this formulation effectively reduced the IOP of glaucomatous rabbits and prolonged the release profile of pilocarpine. Draize’s test demonstrated that these formulations were safe for ocular tissues, with no signs of irritation observed during the study period (Jain et al., 2020).

Aboali et al. (2020) investigated the anti-inflammatory effects of curcumin loaded in niosomal gel on rabbit eyes and found that the formulation reduced inflammation by the same amount produced by marketed corticosteroids (40%) with minimal side effects. Cremophore RH, lecithin, and cholesterol were used as nonionic surfactants in the preparation of pronisomes. These spherical and uniform proniosomes increased the corneal permeation and resistance time. The corneal permeation of proniosomal gel formulation was 3.22-fold higher than curcumin dispersion. The formulation efficiency in lowering ocular pressure and anti-inflammatory effects was evaluated by eye drop instillation every 4 h for 6 days. The measurement of IOP before and after 8 h of instillation indicated that marketed corticosteroids increased the IOP (1.5-fold) more than the curcumin pronisomal gel and curcumin suspension (Aboali et al., 2020).

## 5 Safety of Nanoparticles

Despite many developments of nanotechnology in ophthalmic drug delivery, the fate, toxicity, aggregation, long-term effect, and clearance of nanocarriers are in discussion and a few numbers of these drugs are in the market due to many limitations in their production from *in vitro* testing, *in vivo* animal studies to human studies (due to the difference between corneal mucoadhesion, mucus and tear productions 4 of rabbit eyes and human eyes). There are many factors that influence the toxicity of nanocarriers such as dose of administration, shape, size, surface charge, and functional groups of nanocarriers. So further investigations are needed to ensure the advantages and efficiency of nanocarriers in humans (Maulvi et al., 2021). On the other hand, natural products with numerous benefits such as safety, efficiency, and promising therapeutic effects suffer from low bioavailability, stability, degradation, and elimination. Moreover, most of their superior therapeutic effects in animal models are not the same as the human model in clinical trials.

For determining the safety and toxicity of ophthalmic nanoformulation, the Draize test on rabbit eyes and *in-vivo* test on corneal epithelium cells of the human eye have been performed. For instance, Zhang et al. (2014a) used NLC for ocular delivery of GEN and the Draize test results indicated no toxicity or eye irritation. On the other hand Prow et al. demonstrated the intravitreal injection of chitosan nanoparticles causes irritation of the eye (Prow, 2010). According to these data and other findings, we can summarize that LNPs and liposomes are more suitable, safe, and biocompatible carriers to interact with the biological membrane, use in ocular drug delivery and introduce into the market (Gorantla et al., 2020).

## 6 Discussion

According to mentioned information, the structure, size, composition, and surface properties of nanocarriers have critical effects on corneal permeation and retention of nanoformulations. Particles under 10 µm in size can be better tolerated by the human eye. However, the most suitable size for ocular administration is between 50 and 400 nm provides more effective mucoadhesion and passes through ocular barriers to target the specific site, and causes less ocular irritation (Silva et al., 2021). Furthermore, the surface properties of nanocarriers are another determining factor in ocular drug delivery. Surfactants are commonly used in the production of most nanoparticles to enhance the dissolution and permeation of drugs across cellular membranes. Nonionic surfactants are mostly used in ophthalmic formulations to enhance stability, permeability, solubility, biocompatibility, and decrease the toxicity of nanoformulations. Some of the surfactants are more toxic than others and irritate the eye and should be eliminated once the nanocarriers are formulated. According to Leonardi et al. (2014) findings, some surfactants caused no irritation to the eye such as Kolliphor^®^ P188, and some made irritation in a high concentration such as Tween^®^ 80, and some of them like sodium dodecyl sulfate made severe irritation (Üner et al., 2021). Cationic nanocarriers in comparison to neutral or anionic carriers can bind more effectively to the negatively charged mucin (in corneal and conjunctival epithelium), enhance the retention time, and the therapeutic effect of the entrapped drugs. But the toxicity of cationic LNPs related to surfactants remains a concern. Silva et al. (2019) demonstrated that CTAB in comparison to DDAB is more toxic at the same concentration. Moreover, the structure of polymers or other compounds used in the nanocarriers preparation has a great effect on encapsulation efficiency, drug loading, drug release, and their stability against degrading enzymes, oxidative agents, hydrolysis, or light. Natural compounds can incorporate into the nanocarriers *via* the hydrophobic and electrostatic interactions or hydrogen bonding between natural compounds and polymers which is another determining factor in drug release and storage stability. The phenolic hydroxyl group that exists in flavonoids such as ECGC and catechin is used for fabricating many nanoparticles to enhance stability, biocompatibility, biodegradability, and safety. Polyphenols show a great tendency to polymers *via* noncovalent interactions (Guo et al., 2021). The presence of carbonyl and phenolic groups in the structure of natural compounds can induce noncovalent interactions with other compounds such as polymers. For instance, *β*-Cyclodextrin with a cone-shaped structure is hydrophilic at the outer surface due to the presence of many hydroxyl groups and hydrophobic at its cavity that can encapsulate hydrophobic drugs with suitable size such as curcumin (with phenolic and carbonyl groups) *via* hydrophobic interactions. Yallapu et al. (2010) demonstrated that *β*-cyclodextrin has higher entrapment efficiency in encapsulating curcumin in comparison to PLGA NPs. The hydrophilicity and hydrophobicity of drugs can affect the selection of nanocarriers. For example, in dual drug delivery of BBR (hydrophilic drug) and CHR (hydrophobic drug), the best vehicle to carry these drugs to the target site is liposomes to accommodate CHR in the hydrophilic core and BBR at its hydrophobic shell to control the release of drugs (Lai et al., 2019). The selection of appropriate nanocarrier in ocular drug delivery is directly related to the aim of the study such as the amount of permeation, nature of drugs, kind of ocular disease, and targeted tissues. Overly, among different nanocarriers used in ocular drug delivery, LNPs are delivery systems with both advantages of PNPs and liposomes due to their preparation methods without using toxic organic solvents. Moreover, the production process of liposomes is more expensive and complicated than LNPs production. The small size, stability, possibility to scale up, easy production, the ability of sterilization, increase corneal absorption with enhanced bioavailability and corneal retention time, positive charge of cationic LNPs (Wang et al., 2021), ability to penetrate different parts of ocular tissues especially posterior segment, and lipophilic nature of natural products (such as phenolic compounds), make LNPs as a promising carrier for ocular drug delivery of natural compounds (Faridi Esfanjani et al., 2018).

## 7 Conclusion and Future Perspectives

Ocular diseases as a vision-threatening disorder attracted the attention of scientists due to many challenges in conventional therapies. On the other hand, Natural compounds with many beneficial effects on the treatment and prevention of eye disorders encounter many limitations in their solubility, absorption, and bioavailability. In this review, we discussed the potency of nanotechnology to resolve these limitations and transfer therapeutic natural products to the target site. Nanotechnology merges with pharmaceutics to introduce novel compounds to solve the problems of conventional ocular drug delivery and treatment of ocular disorders. Despite a few documents on the delivery of natural products, the encapsulation of them in nanocarriers enhances their therapeutic effects, bioavailability, stability, efficiency, ocular tolerability, and reduce toxicity. However, the toxicity and scale-up production of these carriers in the industry remain a big challenge so more research on this topic needs to be undertaken for applying these nanoparticles in clinics. The aim of future studies is to enhance the therapeutic effect of natural compounds, drug targeting, bioavailability, safety, and reduce the frequency of administration by reducing the drug’s side effects and enhancing patient compliance. Moreover, drug delivery to the posterior segment of the eye is also challenging, and the most common method for drug delivery to the posterior segment is through intravitreal injection. Researchers aim to develop nanocarriers to overcome the ocular barriers and be helpful in the treatment of ocular diseases, especially those related to the posterior segment with more efficiency, safety, and reduced frequency of administrations.

## 8 Literature Search

A comprehensive search was carried out through the PubMed database on the articles using the combination of different terms in three various fields such as (ophthalmology, eye, ocular, cornea, retina, glaucoma, cataract, chemical burn, corneal injuries, and cornea chemical burns) AND (nano, liposomes, nanofibers, nanoparticles, nanospheres, nanocapsules, hydrogel, chitosan nanoparticles, polymeric nanoparticles, and ocular drug delivery), AND (herbs, natural products, curcumin, quercetin, and the other natural products in a row). For example combination of three terms such as eye AND nano AND curcumin, and the combination of two terms such as curcumin AND ocular drug delivery, etc., more than it, the other electronic databases used and the related articles extruded by applying the aforementioned terms noted in the references list. Only English articles published after 1990 were used, however for historical purposes a limited number of articles were included before this time.
